# Dislocation of the fibular head in an unusual sports injury: a case report

**DOI:** 10.1186/1752-1947-2-158

**Published:** 2008-05-15

**Authors:** Riaz Ahmad, Ruth Case

**Affiliations:** 1 Department of Orthopaedics, Weston General Hospital, Grange Road, Uphill, Weston-Super-Mare BS23 4TQ, UK

## Abstract

**Introduction:**

One of the primary functions of the proximal tibiofibular joint is slight rotation to accommodate rotational stress at the ankle. Proximal tibiofibular joint dislocation is a rare injury and accounts for less than 1% of all knee injuries. This dislocation has been reported in patients who had been engaged in football, ballet dancing, equestrian jumping, parachuting and snowboarding.

**Case presentation:**

A 20-year-old man was injured whilst playing football. He felt a pop in the right knee and was subsequently unable to bear weight on it. The range of movement in his knee joint was limited. Anterior-posterior and lateral X-rays of the knee revealed anterolateral dislocation of the proximal tibiofibular joint. Comparison views confirmed the anterolateral dislocation. He had a failed manipulation under anaesthesia and the joint needed an open reduction in which the fibular head was levered back into place. Operative findings revealed a horizontal type of joint.

**Conclusion:**

An exceedingly rare dislocation of a horizontal type of proximal tibiofibular joint was presented following a football injury. This dislocation was irreducible by a closed method.

## Introduction

One of the primary functions of the proximal tibiofibular joint is slight rotation to accommodate rotational stress at the ankle. Proximal tibiofibular joint dislocation is a rare injury and accounts for less than 1% of all knee injuries. This dislocation has been reported in patients who had been engaged in football, ballet dancing, equestrian jumping, parachuting and snowboarding [1]. It is easily missed on plain radiographs and comparison identical radiographs are necessary to confirm the diagnosis. Knowledge of this injury is not widespread. We describe a rare dislocation of a horizontal type of proximal tibiofibular joint following a football injury. This dislocation was irreducible by a closed method.

## Case presentation

**Figure 1:**
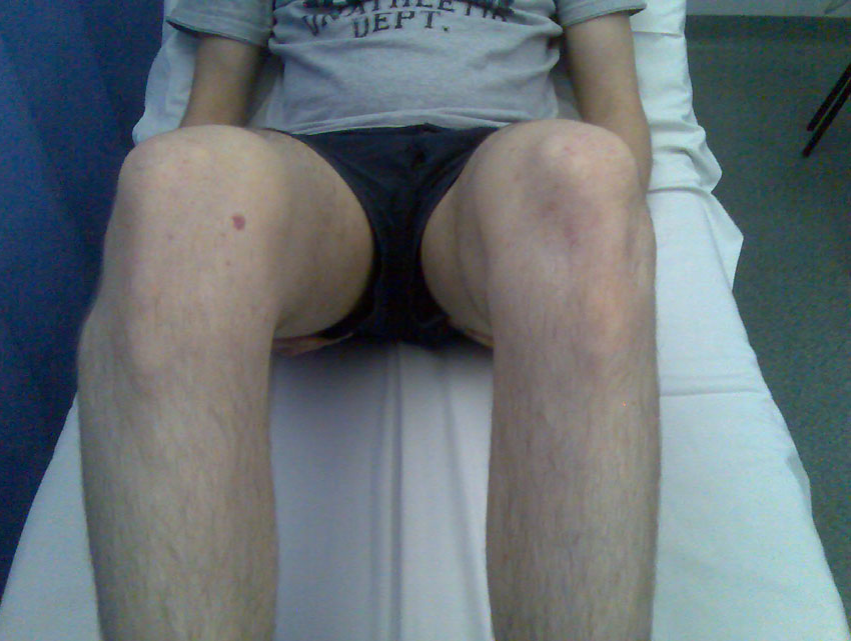
Photograph of the knee showing anterolateral dislocation of the proximal tibiofibular joint.

**Figure 2:**
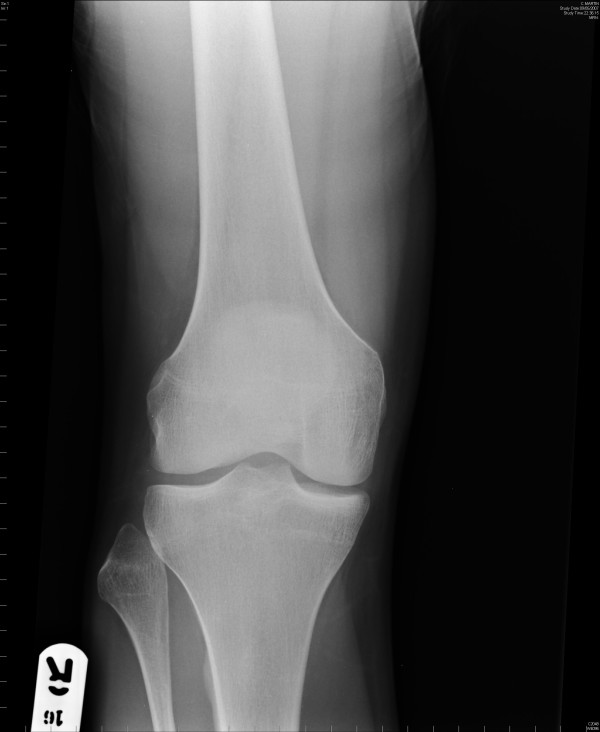
Anterior-posterior view of the knee showing dislocated fibular head.

A 20-year-old man was tackled whilst playing football. He felt a pop in the right knee and was subsequently unable to bear weight on it. He had a palpable bony lump in the anterolateral aspect of his knee and tenderness along the lateral joint line (Figure 1). The range of movement in his knee joint was limited. He had no effusion and there were no signs of ligament injury. Anterior-posterior and lateral X-rays of the knee revealed anterolateral dislocation of the proximal tibiofibular joint (Figure 2). Comparison views confirmed the anterolateral dislocation.

A closed reduction was attempted with the knee flexed, ankle dorsiflexed and everted without any benefit. He also had a failed manipulation under anaesthesia and the joint needed an open reduction in which the fibular head was levered back into place. Operative findings revealed a horizontal type of joint. There was no bone or ligamentous injury. We attribute the failure of the closed reduction to the presence of the projection of the lateral tibial edge as seen in cadaveric study by Ogden [2]. The knee was immobilised for a week and the patient had a good functional outcome.

## Discussion

Anterolateral dislocation is the commonest injury pattern accounting for 85% of dislocations [3]; posteromedial dislocation occurs in 10% and superior dislocation in 2%. The proximal tibiofibular joint is a stable joint with support provided by the joint capsule. The joint is reinforced anteriorly by the biceps femoris tendon insertion into the fibular head, posteriorly by the popliteus tendon, superiorly by the fibular collateral ligament and inferiorly by the interosseous membrane [1].

Knowledge of the anatomical variants of the proximal tibiofibular joint is vital to understand the pathomechanics of the dislocation. Ogden [2] in his study on 50 knees of cadavers described two types of proximal tibiofibular joints: the horizontal and the oblique types with the latter less able to rotate to accommodate torsional stresses and thus commonly associated with dislocations. Moreover, the horizontal articular surface lies behind a projection on the lateral edge of the tibia giving it stability. In Ogden's study [2], 70% of subluxations and dislocations involved oblique joints. Moreover, oblique joints have less articular surface area.

The mechanism of the anterolateral dislocation is inversion and plantar flexion of the ankle that causes tension in the peroneal muscles, extensor digitorum longus and extensor hallucis longus and thus applies a forward dislocating force on the proximal fibula. Flexion of the knee relaxes the biceps tendon and the fibular collateral ligament. Twisting of the body at this point is transmitted along the femur to the tibia, which causes an external rotatory torque of the tibia. Rotatory torque of the tibia along with relaxation of the biceps tendon and collateral ligament causes the fibula to displace laterally while the tensed muscles pull it anteriorly.

A closed reduction should be attempted in patients with acute dislocations. For reduction, the knee should be flexed to relax the biceps tendon and the fibular collateral ligament, the ankle everted and dorsifled to relax the muscles of the anterolateral compartment and external rotatory torque applied to the fibula. Direct pressure applied to the fibula head at this point snaps it back into place. In failed closed reduction, open reduction and stabilisation of the joint can be performed by capsular and ligament repair.

This injury could be easily missed on plain X-rays. Congruity of the proximal tibiofibular joint on lateral views is the key to diagnosis [4]. A comparison view of the normal knee helps in confirmation of the diagnosis. Knowledge of the mechanism of dislocation is vital for a successful closed reduction. Dislocation in a horizontal-type joint variant is rare and as in our case could be a cause of failed closed reduction.

## Conclusion

Dislocation of a horizontal type of proximal tibiofibular joint is exceedingly rare. Proximal tibiofibular dislocation could be mistaken for a meniscal injury due to the nature of the presenting symptoms. This injury is commonly misdiagnosed and could be a potential cause of chronic knee pain and disability.

## Competing interests

The authors declare that they have no competing interests.

## Consent

Written informed consent was obtained from the patient for publication of this case report and any accompanying images. A copy of the written consent is available for review by the Editor-in-Chief of this journal.
